# Muscle‐specific sirtuin 3 overexpression does not attenuate the pathological effects of high‐fat/high‐sucrose feeding but does enhance cardiac SERCA2a activity

**DOI:** 10.14814/phy2.14961

**Published:** 2021-08-17

**Authors:** Christopher J. Oldfield, Teri L. Moffatt, Kimberley A. O'Hara, Bo Xiang, Vernon W. Dolinsky, Todd A. Duhamel

**Affiliations:** ^1^ Faculty of Kinesiology and Recreation Management University of Manitoba Winnipeg MB Canada; ^2^ Institute of Cardiovascular Sciences St. Boniface Hospital Albrechtsen Research Centre Winnipeg MB Canada; ^3^ Department of Pharmacology and Therapeutics Max Rady College of Medicine Rady Faculty of Health Sciences University of Manitoba Winnipeg MB Canada; ^4^ Diabetes Research Envisioned and Accomplished in Manitoba (DREAM) Theme of the Children's Hospital Research Institute of Manitoba Winnipeg MB Canada

**Keywords:** acetylation, calcium handling, diabetes, obesity, SERCA, sirtuins

## Abstract

Obesity, type 2 diabetes, and heart disease are linked to an unhealthy diet. Sarco(endo)plasmic reticulum calcium (Ca^2+^) ATPase 2a (SERCA2a) controls cardiac function by transporting Ca^2+^ in cardiomyocytes. SERCA2a is altered by diet and acetylation, independently; however, it is unknown if diet alters cardiac SERCA2a acetylation. Sirtuin (SIRT) 3 is an enzyme that might preserve health under conditions of macronutrient excess by modulating metabolism via regulating deacetylation of target proteins. Our objectives were to determine if muscle‐specific SIRT3 overexpression attenuates the pathological effects of high fat‐high sucrose (HFHS) feeding and if HFHS feeding alters cardiac SERCA2a acetylation. We also determined if SIRT3 alters cardiac SERCA2a acetylation and regulates cardiac SERCA2a activity. C57BL/6J wild‐type (WT) mice and MCK‐mSIRT3‐M1‐Flag transgenic (SIRT3_TG_) mice, overexpressing SIRT3 in cardiac and skeletal muscle, were fed a standard‐diet or a HFHS‐diet for 4 months. SIRT3_TG_ and WT mice developed obesity, glucose intolerance, cardiac dysfunction, and pathological cardiac remodeling after 4 months of HFHS feeding, indicating muscle‐specific SIRT3 overexpression does not attenuate the pathological effects of HFHS‐feeding. Overall cardiac lysine acetylation was increased by 63% in HFHS‐fed mice (*p* = 0.022), though HFHS feeding did not alter cardiac SERCA2a acetylation. Cardiac SERCA2a acetylation was not altered by SIRT3 overexpression, whereas SERCA2a *V*_max_ was 21% higher in SIRT3_TG_ (*p* = 0.039) than WT mice. This suggests that SIRT3 overexpression enhanced cardiac SERCA2a activity without direct SERCA2a deacetylation. Muscle‐specific SIRT3 overexpression may not prevent the complications associated with an unhealthy diet in mice, but it appears to enhance SERCA2a activity in the mouse heart.

## Graphical abstract

Muscle‐specific SIRT3 overexpression may not prevent the complications associated with an unhealthy diet in mice, but it appears to enhance SERCA2a activity in the mouse heart, independently of direct SERCA2a‐deacetylation.

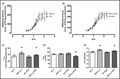

## 1 INTRODUCTION

An unhealthy diet is one of the many factors linked to the development of obesity and type 2 diabetes (T2D) (Leitner et al., 2017). Globally, over 650 million people are obese and approximately 4 million people die from obesity annually (World Health Organization, 2021). Similarly, more than 500 million people are affected by T2D and the number of cases is expected to rise in the future (Kaiser et al., 2018). People with obesity and/or T2D are at an increased risk of developing heart disease because these conditions induce cardiac dysfunction (Alpert et al., 2018; Jia et al., 2018).

Sarco(endo)plasmic reticulum calcium (Ca^2+^) ATPase 2a (SERCA2a) is the primary Ca^2+^ transporter that actively pumps Ca^2+^ from the cytosol, back to the sarcoplasmic reticulum (SR) in cardiomyocytes, facilitating cardiac relaxation (Bers, 1997; Periasamy & Huke, 2001). Ca^2+^ transport by SERCA2a is tightly coupled to ATP hydrolysis because an approximately 10,000‐fold Ca^2+^ concentration gradient is present across the SR membrane at rest (Toyoshima, 2009). In the heart, SERCA2a activity is modulated by many processes (Stammers et al., 2015), including changes in total SERCA2a protein level (Gianni et al., 2005), phospholamban (PLN)‐mediated regulation of SERCA2a (MacLennan & Kranias, 2003), or post‐translational modifications (PTM) that alter SERCA2a enzyme kinetics (Stammers et al., 2015). For example, acetylation of SERCA2a is associated with both enhanced and impaired cardiac SERCA2a activity (Gorski et al., 2019; Meraviglia et al., 2018). Diet‐induced obesity and diabetes are known to modify cardiac SERCA2a protein level and activity (Netticadan et al., 2001; Yao et al., 2015), potentially leading to abnormal Ca^2+^ cycling in cardiomyocytes. Though, it is unknown if an unhealthy diet impacts the acetylation status of SERCA2a in the heart.

Sirtuin (SIRT) proteins are NAD^+^‐dependent histone deacetylases (HDAC) and their activity is integrated with cellular metabolism (Chang & Guarente, 2014). The SIRT family of proteins have garnered considerable attention, as it has been discovered that they have a variety of functions in health and disease, including the potential capacity to extend lifespan in some organisms (Dolinsky, 2017; Grabowska et al., 2017; Matsushima & Sadoshima, 2015). There are seven known SIRT isoforms which are localized to different areas of the cell (Dolinsky, 2017). In cardiomyocytes, SIRT3 is found in the mitochondria (Dolinsky, 2017) where, via deacetylation, it regulates many proteins involved in metabolic control and ATP synthesis (Dolinsky, 2017; Pillai et al., 2010). Evidence also indicates that SIRT3 exists in the nucleus and cytoplasm, where, in response to stress, it can deacetylate proteins and modulate physiological processes outside the mitochondria (Iwahara et al., 2012; Sundaresan et al., 2008). The role of SIRT3 as a regulator of cellular metabolism is demonstrated by SIRT3 deficient mice which are more susceptible to the pathological effects of a diet containing excess fat, as they rapidly develop obesity, insulin resistance, hyperlipidemia, and hepatic steatosis following high fat (HF) feeding (Hirschey et al., 2011). SIRT proteins generally induce favorable changes to energy balance and stimulate the metabolism of lipids and glucose (Kurylowicz, 2016). Thus, SIRT3 has been suggested as a potential therapeutic target for preventing the development of diet‐induced obesity and mitigating its pathological effects, including obesity‐related cardiac dysfunction (Zeng et al., 2015). As well, diminished cardiac SIRT3 protein levels have been reported to contribute to the development of cardiac dysfunction in mice subjected to 24 weeks of high fat‐high sucrose (HFHS) feeding (Kanwal et al., 2019). However, the effects of SIRT3 overexpression in conditions of macronutrient excess have not been examined. Additionally, SIRT1 has been previously reported to deacetylate cardiac SERCA2a and enhance its activity (Gorski et al., 2019; Sulaiman et al., 2010), but it is unclear if other SIRT proteins influence cardiac SERCA2a acetylation or activity.

Therefore, the objectives of this study were to determine if muscle‐specific SIRT3 overexpression attenuates the pathological effects of HFHS feeding and if HFHS feeding alters cardiac SERCA2a acetylation. We also sought to determine if SIRT3 alters cardiac SERCA2a acetylation and regulates cardiac SERCA2a activity.

## 2 MATERIALS AND METHODS

### 2.1 Experimental animals and high‐fat high‐sucrose feeding

The Animal Research: Reporting of In Vivo Experiments (ARRIVE) guidelines 2.0 were adhered to in the development of this manuscript (du Sert et al., 2020). The animal protocol was approved by the University of Manitoba Animal Protocol Management and Review Committee and all animals were treated in accordance with the Canadian Council on Animal Care in Science (2012). Twenty‐four adult (12 weeks of age) male C57BL/6J wild‐type (WT) mice and 24 adult male MCK‐mSIRT3‐M1‐Flag transgenic (SIRT3_TG_) mice overexpressing the full length, murine form of SIRT3 containing a mitochondrial localization signal on a C57BL/6J background were bred in‐house at the Children's Hospital Research Institute of Manitoba by Dr. Vernon Dolinsky's laboratory (University of Manitoba) and utilized in this study. The SIRT3_TG_ mice used to establish the breeding colony were originally obtained from Dr. Qiang Tong's laboratory (Baylor College of Medicine). The overexpression of SIRT3 in these transgenic mice is restricted to cardiac and skeletal muscle using a muscle creatine kinase promoter. One half of the WT mice and one half of the SIRT3_TG_ mice were randomly assigned to groups provided ad libitum access to either a standard diet (Control: catalogue no. 5001, LabDiet) containing 28.05% kcal of protein, 12.14% kcal of fat, and 59.81% kcal of carbohydrates, or a HFHS diet (catalogue no. 12451, Research Diets) containing 20% kcal of protein, 45% kcal of fat, and 17% kcal of sucrose for 4 months. This HFHS feeding model was utilized because it has been previously reported to stimulate the development of obesity, glucose intolerance, and cardiac dysfunction in mice (Pulinilkunnil et al., 2014). The mice were provided free access to drinking water and housed in a conventional mouse housing facility for the duration of the 4‐month study in standard mouse cages, containing wood‐chip bedding (P. J. Murphy Forest Products) on a 12:12‐h light‐dark cycle. Cage assessments occurred daily to ensure the health of mice.

### 2.2 Intraperitoneal glucose tolerance test

Glucose tolerance was assessed using an intraperitoneal glucose tolerance test (IPGTT) at baseline, 3 months, and completion of the 4‐month study. The mice were fasted for 12 h to achieve a baseline blood glucose level before the IPGTT. After the fast, a blood sample was taken from the tail of each mouse at baseline, and then 15, 30, 60, and 120 min following an intraperitoneal injection of glucose (2 g/kg bodyweight). Blood glucose concentrations were determined from blood samples measured using OneTouch^®^ glucose meter (LifeScan). Area under the curve (AUC) of blood glucose concentration was calculated using GraphPad Prism 5 (GraphPad Software).

### 2.3 Echocardiography

Cardiac function and structure were characterized by echocardiography at baseline, 3 months, and completion of the 4‐month study, as previously described (Chowdhury et al., 2017). Noninvasive echocardiography was performed using the Vevo 2100 ultrasound system (FUJIFILM Visualsonics, ON, Canada) with a 17.5 MHz transducer. Mouse body temperature was maintained at 37°C under mild anesthesia (sedated with 3% isoflurane and 1.0 L/min oxygen and maintained at 1%–1.5% isoflurane and 1.0 L/min oxygen) during echocardiography. Parameters were assessed using brightness mode, motion mode, and Doppler imaging. Cardiac function and structural parameters assessed included, left ventricle (LV) weight, heart rate (HR), stroke volume (SV), cardiac output (CO), left ventricular ejection fraction (LVEF), fractional shortening (FS), A wave, E wave, E/A ratio, LV diastolic and systolic diameter (LV Diameter;d; LV Diameter;s), LV diastolic and systolic volume (LV Volume;d; LV Volume;s), left ventricular anterior wall thickness in diastole and systole (LVAW;d; LVAW;s), left ventricular posterior wall thickness in diastole and systole (LVPW;d; LVPW;s), and left ventricular internal dimension in diastole and systole (LVID;d; LVID;s). Echocardiography data were analyzed using Vevo LAB cardiovascular software (FUJIFILM VisualSonics) and measurements were averaged over four cardiac cycles. All images were recorded and analyzed by a trained small animal echocardiographer.

### 2.4 Tissue collection

Mice were removed from their housing cages 2 h before tissue collection at the completion of the 4‐month study. Mice were anesthetized by an intraperitoneal injection of ketamine‐xylazine (150:100 mg/kg) in accordance with the Canadian Council on Animal Care guidelines and the regulations and policies of the University of Manitoba Animal Protocol Management Review Committee (du Sert et al., 2020). The heart of each mouse was rapidly excised, and the LV was isolated. A portion of LV was immediately placed in a microcentrifuge tube, snap‐frozen in liquid nitrogen, and stored at −80°C for use in future experiments. The remaining LV tissue was diluted 1:10 (*w/v*) in ice‐cold phenylmethylsulphonyl fluoride (PMSF) buffer (pH 7.5) containing 250 mM sucrose (Sigma‐Aldrich), 5 mM HEPES (Sigma‐Aldrich), 0.2 mM PMSF (Sigma‐Aldrich), and 0.2% (*w/v*) NaN_3_ (Sigma‐Aldrich), and homogenized using an all‐glass tissue grinder (Kimble Chase Life Sciences) on ice. LV tissue homogenate was then aliquoted into microcentrifuge tubes, snap frozen in liquid nitrogen, and stored at −80°C for future use in SERCA2a activity and western blot analyses. The gastrocnemius muscle was isolated from six mice per group and homogenized to confirm SIRT3 overexpression in the skeletal muscle of SIRT3_TG_ mice with western blotting. The liver of each mouse was rapidly excised and weighed. The livers from five mice per group were homogenized and SIRT3 protein levels were measured by western blotting to confirm that SIRT3 protein levels were not altered in this non‐muscle tissue of SIRT3_TG_ mice. The total protein content of each tissue homogenate sample was determined in duplicate with the Detergent Compatible™ Protein Assay (Bio‐Rad Laboratories) using bovine serum albumin (Sigma‐Aldrich) (BSA) as the protein standard.

### 2.5 Mitochondrial fractionation

The mitochondrial fraction was isolated from the LV tissue of two mice per group using the Mitochondrial Isolation Kit for Tissue (Fisher Scientific) according to the manufacturer's protocol. The mitochondrial pellet was then lysed with ice‐cold cell lysis buffer (New England Biolabs) and western blotting was used to measure mitochondrial lysine acetylation.

### 2.6 Co‐immunoprecipitation

The Signal‐Seeker™ Acetyl‐Lysine detection kit (Cytoskeleton) was used to measure SERCA2a acetylation in LV tissue according to the manufacturer's protocol. LV tissue was removed from −80°C and immediately lysed with ice‐cold BlastR™ lysis buffer (Cytoskeleton) and DNA was removed from the lysate using the BlastR™ filter system (Cytoskeleton). Following dilution with BlastR™ dilution buffer (Cytoskeleton), the protein concentration of the lysate was determined with the Precision Red™ Protein Assay Reagent (Cytoskeleton) and assessed at 600 nm. The sample was then immunoprecipitated by incubating the lysate with acetylated‐lysine affinity beads (Cytoskeleton) overnight at 4°C with rotation. The beads were pelleted and washed 3 times with BlastR™ wash buffer. Bound acetylated‐lysine containing proteins were eluted using bead elution buffer (Cytoskeleton) and acetylated‐SERCA2a was detected on polyvinylidene difluoride (PVDF) membranes (Bio‐Rad Laboratories) by western blotting with an anti‐SERCA2a primary antibody (catalogue no. 4388, Cell Signaling Technology; 1:1000). The total amount of immunoprecipitated acetylated‐lysine containing proteins was detected on PVDF membranes (Bio‐Rad Laboratories) by western blotting with an anti‐acetylated‐lysine antibody conjugated to horseradish peroxidase (catalogue no. 19C4B2.1, Cytoskeleton; 1:3000). The total level of acetylated‐SERCA2a was normalized to the total level of acetylated‐lysine immunoprecipitated and expressed as a percentage of WT‐C. Acetylated‐SERCA2a was measured in LV tissue from six mice per group by co‐immunoprecipitation.

### 2.7 SERCA2a activity

SERCA2a activity was measured in LV tissue homogenate using a spectrophotometric assay previously described by Simonides and van Hardeveld (Simonides & Hardeveld, 1990) and modified by Duhamel et al. (Duhamel et al., 2007). SERCA2a ATP hydrolysis was measured over Ca^2+^ concentrations ([Ca^2+^]) ranging from a pCa (−log_10_([Ca^2+^])) of 7.66–5.76 by assessing the rate of NADH disappearance at 340 nm and 37°C for 30 min using a FLUOstar^®^ Omega microplate reader (BMG Labtech, GER). The reaction buffer (pH 7.0) contained 200 mM KCl (Fisher Scientific), 20 mM HEPES (Sigma‐Aldrich), 15 mM MgCl_2_ (Fisher Scientific), 10 mM NaN_3_ (Sigma‐Aldrich), 10 mM phosphoenolpyruvate (Roche Applied Science, GER), 5 mM ATP (Sigma‐Aldrich), and 1 mM EGTA (Sigma‐Aldrich). The SERCA‐specific inhibitor, cyclopiazonic acid (Seidler et al., 1989) (Sigma‐Aldrich) (CPA; 40 mM) was added to one reaction for each sample to determine the basal ATPase activity. SERCA2a activity was calculated based on the difference in the rate of ATP hydrolysis stimulated by Ca^2+^ in the absence and presence of CPA. The data for each sample were plotted to create a graph of SERCA2a activity versus pCa values using GraphPad Prism 5 (GraphPad Software). The following Ca^2+^‐dependent kinetic properties of SERCA2a were then calculated: (1) *V*_max_, the maximal enzyme activity; (2) Ca_50_, the [Ca^2+^] eliciting 50% of *V*_max_; (3) Hill coefficient, the slope of the relationship between [Ca^2+^] and enzyme activity for 10%–90% of *V*_max_. SERCA2a activity in LV tissue homogenate was measured in duplicate for all mice.

### 2.8 Western blotting

Thirty microgram of protein was diluted and denatured in 2x Laemmli sample buffer (Bio‐Rad Laboratories). Samples were heated at 95°C for 5 min and underwent SDS‐PAGE using Bio‐Rad 4%–15% Mini‐PROTEAN^®^ TGX Stain‐Free™ precast gels (Bio‐Rad Laboratories). Gels were activated using the Bio‐Rad ChemiDoc™ MP imager (Bio‐Rad Laboratories) after electrophoresis. This was followed by semi‐dry transfer onto nitrocellulose or PVDF membranes (Bio‐Rad Laboratories) using the Bio‐Rad Trans‐Blot^®^ Turbo™ transfer system (Bio‐Rad Laboratories). Membranes were then blocked with 5% (*w/v*) BSA (Sigma‐Aldrich) in Tris‐buffered saline (pH 7.5) with 0.1% Tween^®^ 20 (Sigma‐Aldrich) (TBST) buffer for 1 h at room temperature. Blots were incubated overnight at 4°C with the following primary antibodies diluted in 5% BSA (Sigma‐Aldrich) in TBST buffer: anti‐SERCA2a (catalogue no. 4388, Cell Signaling Technology; 1:1000), anti‐acetylated‐lysine (Co‐immunoprecipitation experiments; catalogue no. 19C4B2.1, Cytoskeleton; 1:3000), anti‐SIRT3 (catalogue no. 5490, Cell Signaling Technology; 1:1000), anti‐acetylated‐lysine (catalogue no. 9441, Cell Signaling Technology; 1:500), anti‐PLN (catalogue no. 14562, Cell Signaling Technology; 1:1000), anti‐phosphorylated‐PLN^Ser16/Thr17^ (catalogue no. 8496, Cell Signaling Technology; 1:1000), anti‐NCX1 (catalogue no. R3F1, Swant, SUI; 1:1000), anti‐Calsequestrin (catalogue no. ab3516, Abcam; 1:1000), anti‐AMPKα (catalogue no. 2532, Cell Signaling Technology; 1:1000), anti‐phosphorylated‐AMPKα^Thr172^ (catalogue no. 4188, Cell Signaling Technology; 1:1000), and anti‐TFB2M (catalogue no. 24411–1‐AP, PTG Labs; 1:1000). Blots were washed for 10 min, 3 times, with TBST. Membranes were incubated in anti‐rabbit secondary antibody conjugated to horseradish peroxidase (catalogue no. 7074, Cell Signaling Technology) diluted 1:5000 in 5% BSA (Sigma‐Aldrich) in TBST buffer for 2 h at room temperature and then washed for 10 min, 3 times, with TBST. Proteins were visualized using Clarity™ Western enhanced chemiluminescent substrate (Bio‐Rad Laboratories) and the Bio‐Rad ChemiDoc™ MP imager (Bio‐Rad Laboratories). The specific conditions for the detection of each protein by western blotting are described in Table 1. All images were analyzed using Image Lab software (Bio‐Rad Laboratories). Relative protein levels were determined by normalization to total protein and expressed as a percentage of WT‐C. Phosphorylated‐AMPKα^Thr172^ and phosphorylated‐PLN^Ser16/Thr17^ were normalized to total‐AMPKα and total‐PLN, respectively, to determine the phosphorylated protein to total protein ratios. LV tissue from 6 to 10 mice per group was analyzed by western blotting.

**TABLE 1:** The gel, membrane, blocking agent, primary antibody conditions, secondary antibody conditions, and enhanced chemiluminescent substrate used for the detection of each protein by western blotting

Protein	Gel	Membrane	Blocking agent	Primary antibody (Time/Temperature)	Secondary antibody (Time/Temperature)	ECL substrate
Acetylated‐SERCA2a (co‐IP)	4%–15% Gradient	PVDF	5% BSA in TBST	1:1000 (ON/4°)	1:1000 (1 h/RT)	Clarity™
Acetylated‐Lysine (co‐IP)	4%–15% Gradient	PVDF	5% BSA in TBST	1:3000 (ON/4°)	‐	Clarity™
SIRT3	4%–15% Gradient	Nitrocellulose	5% BSA in TBST	1:1000 (ON/4°)	1:1000 (1 h/RT)	Clarity™
Acetylated‐Lysine	4%–15% Gradient	Nitrocellulose	5% BSA in TBST	1:500 (ON/4°)	1:1000 (1 h/RT)	Clarity™
SERCA2a	4%–15% Gradient	Nitrocellulose	5% BSA in TBST	1:1000 (ON/4°)	1:1000 (1 h/RT)	Clarity™
PLN	4%–15% Gradient	Nitrocellulose	5% BSA in TBST	1:1000 (ON/4°)	1:1000 (1 h/RT)	Clarity™
Phosphorylated‐PLN	4%–15% Gradient	Nitrocellulose	5% BSA in TBST	1:1000 (ON/4°)	1:1000 (1 h/RT)	Clarity™
NCX1	4%–15% Gradient	Nitrocellulose	5% BSA in TBST	1:1000 (ON/4°)	1:1000 (1 h/RT)	Clarity™
Calsequestrin	4%–15% Gradient	Nitrocellulose	5% BSA in TBST	1:1000 (ON/4°)	1:1000 (1 h/RT)	Clarity™
AMPKɑ	4%–15% Gradient	Nitrocellulose	5% BSA in TBST	1:1000 (ON/4°)	1:1000 (1 h/RT)	Clarity™
P‐AMPKɑ	4%–15% Gradient	Nitrocellulose	5% BSA in TBST	1:1000 (ON/4°)	1:1000 (1 h/RT)	Clarity™
TFB2 M	4%–15% Gradient	Nitrocellulose	5% BSA in TBST	1:1000 (ON/4°)	1:1000 (1 h/RT)	Clarity™

Abbreviations: BSA, bovine serum albumin; co‐IP, co‐immunoprecipitation; ECL, enhanced chemiluminescent; ON, overnight; PVDF, polyvinylidene difluoride; RT, room temperature; TBST, Tris‐buffered saline with 0.1% Tween^®^ 20.

### 2.9 Statistical analyses

Two‐way and two‐way repeated measures analysis of variance were used to detect differences between groups based on genotype, diet, and their interaction. A Tukey post hoc test was used to identify differences between specific means when significant difference (*p* ≤ 0.05) was found. Statistical calculations were made using SPSS version 26 (IBM Corporation). This study was powered using SERCA2a *V*_max_ as its primary outcome variable, indicating a total sample size of 28 (7 mice per group) was needed to detect differences between groups (two tailed ɑ = 0.05; β = 0.20; Mean ± SD, Group 1, 107 ± 21; Group 2, 71 ± 17).

## 3 RESULTS

### 3.1 High fat‐high sucrose‐feeding induced obesity and glucose intolerance

**FIGURE 1:**
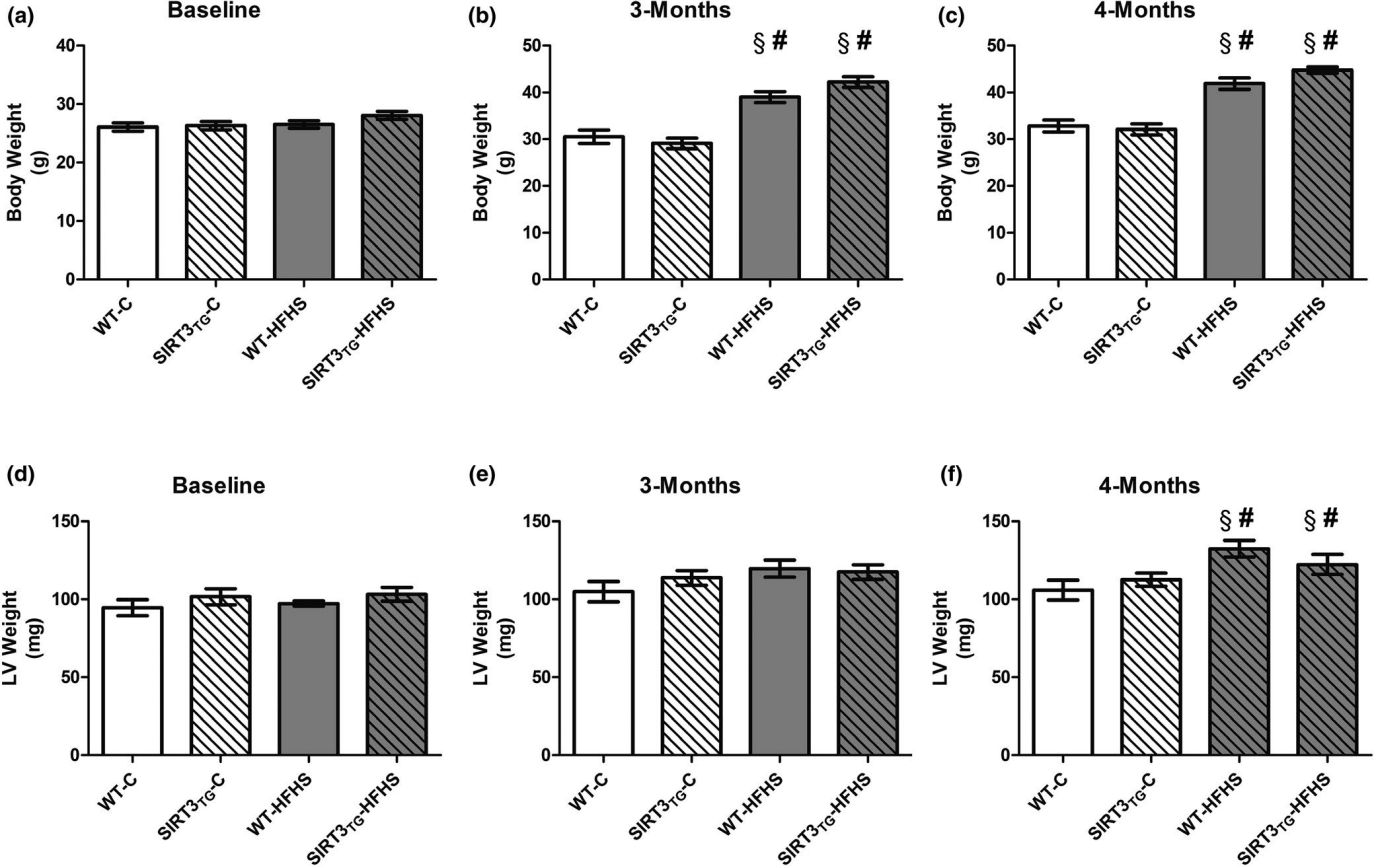
Body weights and LV weights of WT or SIRT3TG mice at baseline and following 3‐ and 4‐months of control‐ or HFHS‐feeding. ^§^ indicates significantly different from baseline (*p* ≤ 0.05). ^#^ indicates a main effect of diet (*p* ≤ 0.05). Compared using a two‐way and two‐way repeated measures ANOVA and a Tukey post‐hoc test (*n* = 12 mice per group). Graphs are presented as the mean ± SE

**FIGURE 2:**
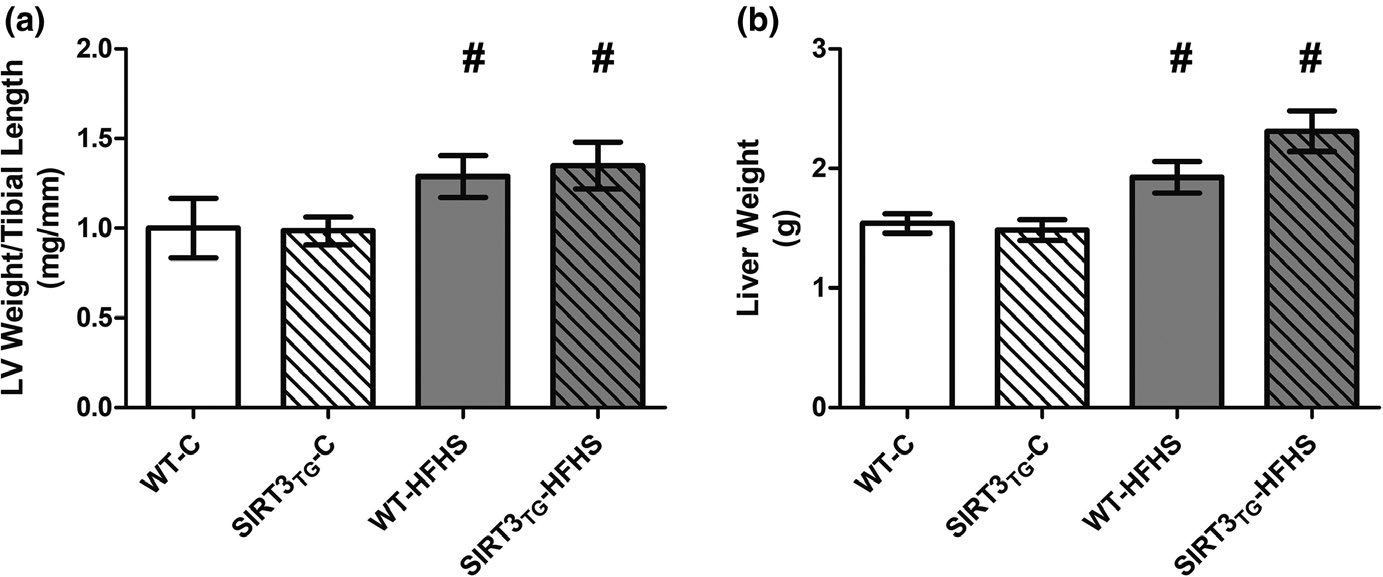
LV weight to tibial Length ratio and liver weights of WT or SIRT3TG mice following 4‐months control‐ or HFHS‐feeding. ^#^ indicates a main effect of diet (*p* ≤ 0.05). Compared using a two‐way ANOVA and a Tukey post‐hoc test (*n* = 12 mice per group). Graphs are presented as the mean ± SE

Muscle‐specific SIRT3 overexpression did not influence mouse body weights (Figure 1a–c). The body weights of mice subjected to HFHS feeding were increased by 49% after 3 months (main effect of diet; *p* ≤ 0.0001) and by 59% after 4 months (main effect of diet; *p* ≤ 0.0001), compared to baseline (Figure 1b,c). The body weights of HFHS‐fed mice were 36% higher than control‐fed mice at 3 months (main effect of diet; *p* ≤ 0.0001), and 34% higher than control‐fed mice at 4 months (main effect of diet; *p* ≤ 0.0001) (Figure 1b,c). There were no differences in the LV weight of SIRT3_TG_ mice, compared to WT mice (Figure 1d–f). The LV weights of HFHS‐fed mice were increased by 27% after 4 months, compared to baseline (main effect of diet; *p* = 0.002) (Figure 1f). The LV weights of HFHS‐fed mice were 17% higher than control‐fed mice at 4 months (main effect of diet; *p* = 0.003) (Figure 1f). After normalization to body size, the LV weights of HFHS‐fed mice remained elevated when compared to control‐fed mice, as the LV weight to tibial length ratio of HFHS‐fed mice was 25% higher at 4 months, compared to control‐fed mice (main effect of diet; *p* = 0.005) (Figure 2a). Muscle‐specific SIRT3 overexpression did not influence mouse liver weights (Figure 2b). The liver weights of HFHS‐fed mice were 40% higher, compared to control‐fed mice at 4 months (main effect of diet; *p* ≤ 0.0001) (Figure 2b).

**FIGURE 3:**
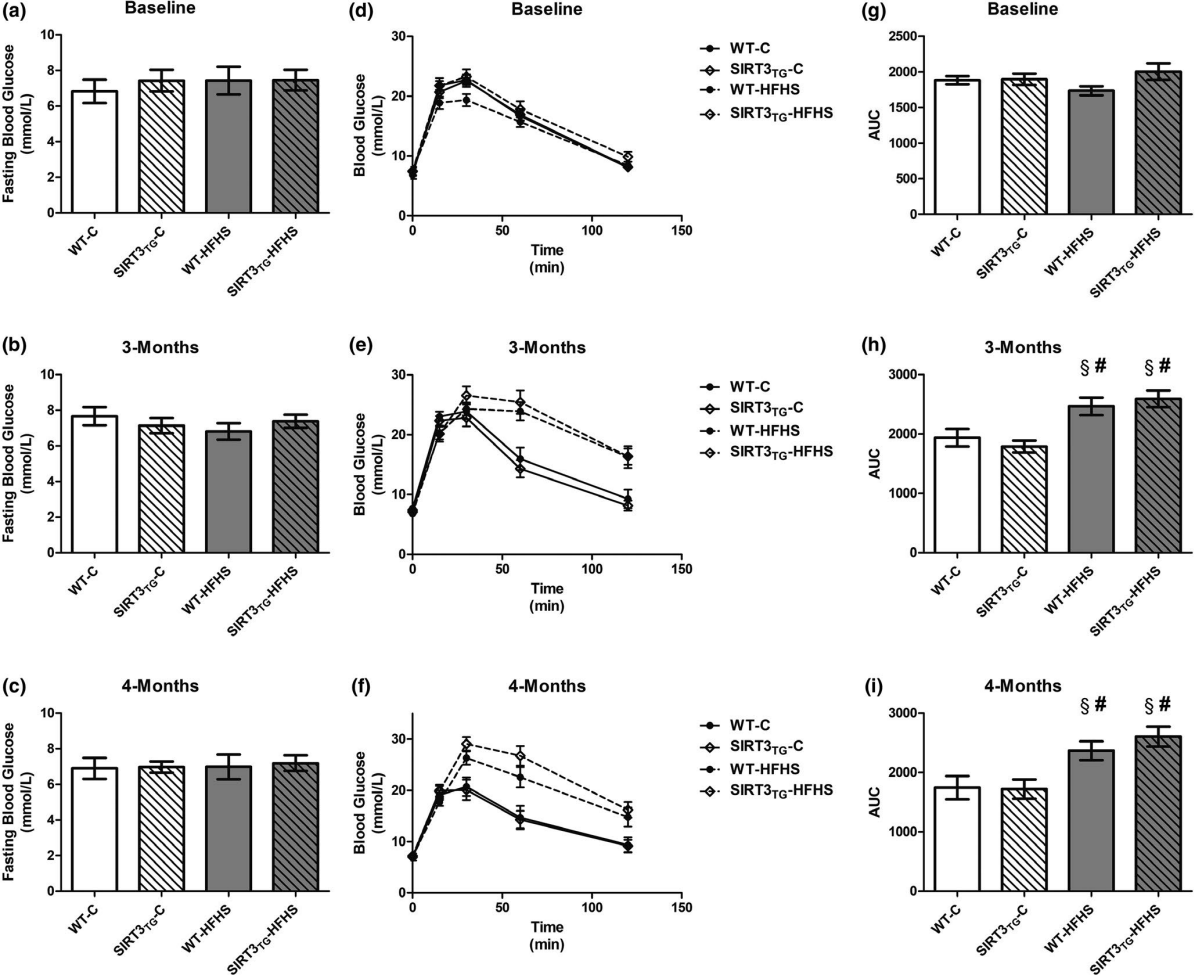
Fasting blood glucose levels, blood glucose levels at baseline, and then 15, 30, 60, and 120 min after an intraperitoneal injection of glucose, and the AUC of blood glucose levels after an intraperitoneal injection of glucose of WT or SIRT3TG mice at baseline and following 3‐ and 4‐months of control‐ or HFHS‐feeding. ^§^ indicates significantly different from baseline (*p* ≤ 0.05). ^#^ indicates a main effect of diet (*p* ≤ 0.05). Compared using a two‐way and two‐way repeated measures ANOVA and a Tukey post‐hoc test (*n* = 12 mice per group). Graphs are presented as the mean ± SE

An IPGTT was used to assess glucose tolerance in WT and SIRT3_TG_ mice at baseline, and then following 3 months and 4 months of either control or HFHS feeding (Figure 3). There were no differences in fasting blood glucose concentrations between SIRT3_TG_ and WT mice, measured prior to an intraperitoneal injection of glucose (Figure 3a–c). No differences in fasting blood glucose concentrations were identified between HFHS‐ and control‐fed mice (Figure 3a–c). Following an intraperitoneal injection of glucose, the AUC of blood glucose concentrations of HFHS‐fed mice were elevated by 35% after 3 months (main effect of diet; *p* ≤ 0.0001) and elevated by 33% after 4 months (main effect of diet; *p* ≤ 0.0001), compared to baseline (Figure 3h,i). The AUC of blood glucose concentrations following an intraperitoneal injection of glucose was 36% higher in HFHS‐fed mice, compared to control‐fed mice at 3 months (main effect of diet; *p* ≤ 0.0001), and 44% higher in HFHS‐fed mice, compared to control‐fed mice a 4 months (main effect of diet; *p* ≤ 0.0001) (Figure 3h,i).

### 3.2 High‐fat high‐sucrose feeding altered cardiac function and structure

There were no significant differences in cardiac functional parameters between SIRT3_TG_ mice and WT mice (Table 2). However, there was a trend of increased SV in SIRT3_TG_ mice, compared to WT mice at 3 months (*p* = 0.074) and at 4 months (*p* = 0.059) (Table 2). Muscle‐specific SIRT3 overexpression altered cardiac structural parameters, as the LVPW;d of SIRT3_TG_ mice was 7% thinner than WT mice at 4 months (main effect of genotype; *p* = 0.031), and the LVID;d of SIRT3_TG_ mice was 4% thicker than WT mice at 4 months (main effect of genotype; *p* = 0.047) (Table 2). At baseline, FS in control‐fed mice was 11% higher, compared to HFHS‐fed mice (main effect of diet; *p* = 0.044) (Table 2). Though this difference did not persist as there were no significant differences in FS between control‐ and HFHS‐fed mice at 3 months (*p* = 0.886) or at 4 months (*p* = 0.315) (Table 2). The A wave forms of HFHS‐fed mice were slowed by 20% after 3 months, compared to baseline (main effect of diet; *p* = 0.003) (Table 2). The A wave forms of HFHS‐fed mice were also 21% slower than control‐fed mice at 3 months (main effect of diet; *p* ≤ 0.0001), and 11% slower than control‐fed mice at 4 months (main effect of diet; *p* = 0.030) (Table 2). The E wave forms of HFHS‐fed mice were slowed by 20% after 3 months (main effect of diet; *p* = 0.004), and slowed by 14% after 4 months, compared to baseline (main effect of diet; *p* = 0.011) (Table 2). The E wave forms of HFHS‐fed mice were 22% slower at 3 months (main effect of diet; *p* = 0.001), and 20% slower at 4 months (main effect of diet; *p* = 0.004), compared to control‐fed mice (Table 2). The LVAW;d of HFHS‐fed mice was thickened by 23% after 3 months (main effect of diet; *p* = 0.008) and thickened by 10% after 4 months (*p* = 0.028), compared to baseline (Table 2). The LVAW;d of HFHS‐fed mice was 18% thicker than control‐fed mice at 4 months (main effect of diet; *p* ≤ 0.0001) (Table 2). The LVAW;s of HFHS‐fed mice was thickened by 14% after 3 months, compared to baseline (main effect of diet; *p* = 0.035). The LVAW;s of HFHS‐fed mice was 12% thicker than control‐fed mice at 4 months (main effect of diet; *p* ≤ 0.001) (Table 2). After 4 months, the LVPW;s of HFHS‐fed mice was 13% thicker, compared to baseline (main effect of diet; *p* = 0.031) (Table 2). An interaction between SIRT3 overexpression and HFHS feeding was identified for the LVAW;d, as the LVAW;d of SIRT3_TG_‐HFHS‐fed mice was 12% thinner than WT‐HFHS‐fed mice at 4 months (*p* = 0.031) (Table 2).

**TABLE 2:** Cardiac structural and functional parameters assessed by echocardiography of WT or SIRT3_TG_ mice at baseline and following 3 and 4 months of control‐ or HFHS‐feeding

	Baseline	3 Months	4 Months
HR (bpm)			
WT‐C	460.19 (16.47)	445.88 (15.17)	490.54 (19.28)
SIRT3_TG_‐C	464.21 (11.21)	457.65 (8.57)	512.84 (13.64)
WT‐HFHS	469.06 (15.47)	471.5 (15.73)	496.58 (11.67)
SIRT3_TG_‐HFHS	452.15 (15.58)	444.45 (17.04)	462.78 (11.61)
SV (uL)			
WT‐C	43.84 (2.51)	47.15 (1.30)	48.09 (2.23)
SIRT3_TG_‐C	48.34 (3.04)	48.31 (2.08)	50.70 (3.37)
WT‐HFHS	44.47 (1.21)	43.31 (2.42)	44.17 (1.46)
SIRT3_TG_‐HFHS	49.26 (2.51)	49.12 (1.51)	50.21 (1.53)
CO (ml/min)			
WT‐C	20.27 (1.50)	21.72 (1.19)	23.82 (1.86)
SIRT3_TG_‐C	22.37 (1.42)	22.98 (0.84)	25.90 (1.72)
WT‐HFHS	20.82 (0.80)	21.34 (1.49)	21.93 (0.86)
SIRT3_TG_‐HFHS	22.54 (1.73)	21.99 (1.39)	23.24 (0.95)
LVEF (%)			
WT‐C	53.18 (2.40)	54.99 (1.99)	50.85 (1.78)
SIRT3_TG_‐C	54.86 (2.61)	52.34 (2.09)	52.79 (2.06)
WT‐HFHS	49.29 (1.85)	50.35 (2.94)	50.54 (2.07)
SIRT3_TG_‐HFHS	50.55 (1.20)	50.6 (2.30)	48.91 (2.17)
FS (%)			
WT‐C	27.35 (1.55)	27.78 (1.22)	25.85 (1.12)
SIRT3_TG_‐C	28.56 (1.76)	28.21 (1.01)	27.15 (1.28)
WT‐HFHS	24.82 (1.12)^d^	26.9 (1.64)	25.65 (1.26)
SIRT3_TG_‐HFHS	25.61 (0.76)^d^	28.71 (1.33)	24.78 (1.36)
A wave (mm/sec)			
WT‐C	–21.61 (1.45)	–22.79 (1.00)	–22.21 (0.82)
SIRT3_TG_‐C	–23.42 (0.98)	–22.52 (1.42)	–22.19 (1.57)
WT‐HFHS	–22.45 (1.06)	–18.08 (0.72)^a^, ^d^	–19.88 (0.92)^d^
SIRT3_TG_‐HFHS	–22.13 (1.55)	–17.61 (0.66)^a^, ^d^	–19.76 (0.82)^d^
E wave (mm/sec)			
WT‐C	–21.36 (1.26)	–23.00 (1.65)	–23.79 (1.78)
SIRT3_TG_‐C	–22.68 (1.03)	–24.16 (1.88)	–26.46 (2.16)
WT‐HFHS	–23.43 (1.70)	–18.62 (0.93)^a^, ^d^	–20.80 (1.50)^a^, ^d^
SIRT3_TG_‐HFHS	–22.45 (2.03)	–17.93 (1.23)^a^, ^d^	–19.62 (0.99)^a^, ^d^
E wave/A wave			
WT‐C	1.00 (0.04)	1.00 (0.05)	1.07 (0.07)
SIRT3_TG_‐C	0.97 (0.04)	1.09 (0.08)	1.20 (0.06)
WT‐HFHS	1.05 (0.06)	1.04 (0.05)	1.04 (0.06)
SIRT3_TG_‐HFHS	1.02 (0.05)	1.02 (0.05)	1.00 (0.05)
LV Diameter;d (mm)			
WT‐C	4.25 (0.10)	4.41 (0.09)	4.43 (0.09)
SIRT3_TG_‐C	4.32 (0.13)	4.43 (0.10)	4.53 (0.14)
WT‐HFHS	4.33 (0.06)	4.30 (0.08)	4.31 (0.07)
SIRT3_TG_‐HFHS	4.47 (0.07)	4.44 (0.07)	4.54 (0.08)
LV Diameter;s (mm)			
WT‐C	3.08 (0.08)	3.19 (0.11)	3.20 (0.08)
SIRT3_TG_‐C	3.03 (0.13)	3.19 (0.10)	3.28 (0.11)
WT‐HFHS	3.17 (0.06)	3.15 (0.11)	3.15 (0.07)
SIRT3_TG_‐HFHS	3.23 (0.05)	3.17 (0.10)	3.29 (0.09)
LV Volume;d (ul)			
WT‐C	81.52 (4.50)	88.53 (3.93)	89.41 (4.47)
SIRT3_TG_‐C	85.34 (6.12)	89.72 (4.57)	94.78 (6.69)
WT‐HFHS	84.73 (2.74)	83.58 (3.54)	83.90 (3.08)
SIRT3_TG_‐HFHS	91.41 (3.49)	89.78 (3.27)	94.72 (3.83)
LV Volume;s (ul)			
WT‐C	37.69 (2.43)	41.38 (3.14)	41.32 (2.67)
SIRT3_TG_‐C	37.00 (3.49)	41.41 (3.06)	44.39 (3.48)
WT‐HFHS	40.26 (1.89)	40.28 (3.53)	39.74 (2.25)
SIRT3_TG_‐HFHS	42.16 (1.72)	40.66 (2.87)	44.51 (3.05)
LVAW;d (mm)			
WT‐C	0.69 (0.04)	0.77 (0.03)	0.76 (0.04)
SIRT3_TG_‐C	0.74 (0.03)	0.82 (0.03)	0.80 (0.03)
WT‐HFHS	0.75 (0.03)	0.83 (0.03)	0.98 (0.03)^a^, ^b^, ^d^
SIRT3_TG_‐HFHS	0.74 (0.03)	0.83 (0.03)	0.86 (0.04)^a^, ^b^, ^d^, ^e^
LVAW;s (mm)			
WT‐C	1.04 (0.05)	1.14 (0.03)	1.10 (0.04)
SIRT3_TG_‐C	1.08 (0.04)	1.13 (0.02)	1.14 (0.04)
WT‐HFHS	1.11 (0.03)	1.17 (0.05)	1.29 (0.03)^a^, ^d^
SIRT3_TG_‐HFHS	1.08 (0.04)	1.18 (0.03)	1.21 (0.05)^a^, ^d^
LVPW;d (mm)			
WT‐C	0.71 (0.04)	0.70 (0.02)	0.75 (0.02)
SIRT3_TG_‐C	0.69 (0.02)	0.70 (0.03)	0.74 (0.02)^c^
WT‐HFHS	0.70 (0.02)	0.74 (0.02)	0.84 (0.02)
SIRT3_TG_‐HFHS	0.71 (0.02)	0.72 (0.02)	0.74 (0.03)^c^
LVPW;s (mm)			
WT‐C	1.01 (0.05)	1.01 (0.02)	1.03 (0.03)
SIRT3_TG_‐C	1.02 (0.03)	1.01 (0.04)	1.08 (0.03)
WT‐HFHS	0.99 (0.03)	1.03 (0.05)	1.15 (0.04)^a^
SIRT3_TG_‐HFHS	1.00 (0.03)	1.03 (0.03)	1.08 (0.04)^a^
LVID;d (mm)			
WT‐C	4.27 (0.09)	4.40 (0.10)	4.42 (0.08)
SIRT3_TG_‐C	4.33 (0.11)	4.43 (0.10)	4.52 (0.13)^c^
WT‐HFHS	4.28 (0.05)	4.31 (0.09)	4.25 (0.08)
SIRT3_TG_‐HFHS	4.42 (0.06)	4.40 (0.05)	4.52 (0.07)^c^
LVID;s (mm)			
WT‐C	3.10 (0.10)	3.14 (0.10)	3.28 (0.09)
SIRT3_TG_‐C	3.10 (0.13)	3.25 (0.12)	3.30 (0.11)
WT‐HFHS	3.22 (0.08)	3.21 (0.11)	3.16 (0.08)
SIRT3_TG_‐HFHS	3.29 (0.05)	3.27 (0.09)	3.41 (0.10)

Abbreviations: CO, cardiac output; FS, fractional shortening; HR, heart rate; LVAW;d, left ventricular anterior wall thickness in diastole; LVAW;s, left ventricular anterior wall thickness in systole; LV Diameter;d, left ventricular diastolic diameter; LV Diameter;s, left ventricular systolic diameter; LVEF, left ventricular ejection fraction, LVID;d, left ventricular internal dimension in systole; LVID;s, left ventricular internal dimension in diastole; LVPW;d, left ventricular posterior wall thickness in diastole; LVPW;s, left ventricular posterior wall thickness in systole; LV Volume;d, left ventricular diastolic volume; LV Volume;s, left ventricular systolic volume; SV, stroke volume.

^a^ indicates significantly different from baseline (*p* ≤ 0.05).

^b^ indicates significantly different from 3‐months (*p* ≤ 0.05).

^c^ indicates a main effect of genotype (*p* ≤ 0.05).

^d^ indicates a main effect of diet (*P* ≤ 0.05).

^e^ indicates an interaction between genotype and diet (*p* ≤ 0.05). Compared using a two‐way and two‐way repeated measures analysis of variance and a Tukey post hoc test (*n* = 12 mice per group).

### 3.3 High‐fat high‐sucrose feeding and SIRT3 overexpression modulate cardiac lysine acetylation

**FIGURE 4:**
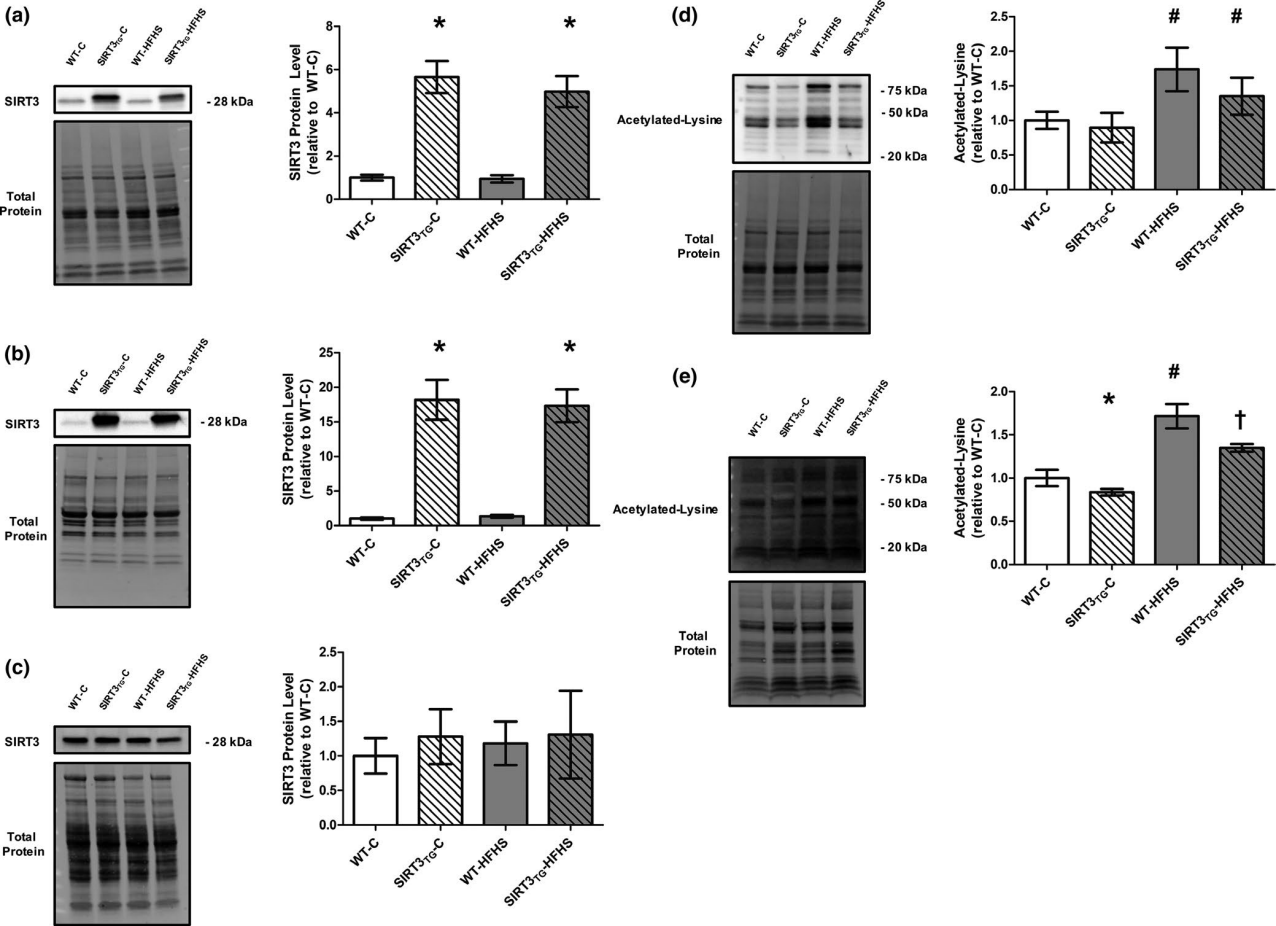
Representative Western blots and graphs depicting LV, gastrocnemius muscle, and liver SIRT3 protein levels and overall and mitochondrial LV acetylated‐lysine protein level from WT or SIRT3TG mice following 4‐months of control‐ or HFHS‐feeding. (a) LV SIRT3. (b) Gastrocnemius SIRT3. (c) Liver SIRT3. (d) Overall Acetylated‐Lysine. (e) Mitochondrial Acetylated‐Lysine. * indicates a main effect of genotype (*p* ≤ 0.05). ^#^ indicates a main effect of diet (*p* ≤ 0.05). Compared using a two‐way ANOVA and a Tukey posthoc test (LV SIRT3: *n* = 10 mice per group; Gastrocnemius SIRT3: *n* = 6 mice per group; Liver SIRT3: *n* = 5 mice per group; Overall Acetylated‐Lysine: *n* = 6 mice per group; Mitochondrial Acetylated‐Lysine: *n* = 2 mice per group). Graphs are presented as the mean ± SE

The muscle‐specific overexpression of SIRT3 in the cardiac and skeletal muscle of SIRT3_TG_ mice was confirmed as SIRT3 protein levels were increased by 5.5‐fold in the LV of SIRT3_TG_ mice, compared to WT mice (main effect of genotype; *p* ≤ 0.0001) (Figure 4a), and SIRT3 protein levels were increased by 15‐fold in the gastrocnemius muscle of SIRT3_TG_ mice, compared to WT mice (main effect of genotype; *p* ≤ 0.0001) (Figure 4b). No differences in liver SIRT3 protein levels were found between WT and SIRT3_TG_ mice, indicating SIRT3 overexpression was not present in this non‐muscle tissue of SIRT3_TG_ mice (Figure 4c). HFHS feeding did not alter LV, gastrocnemius, or liver SIRT3 protein levels (Figure 4a–c). Overall lysine acetylation in LV tissue was unchanged in SIRT3_TG_ mice, compared to WT mice (Figure 4d); whereas overall lysine acetylation was increased by 63% in the LVs of HFHS‐fed mice, compared to control‐fed mice (main effect of diet; *p* = 0.022) (Figure 4d). An interaction between SIRT3 overexpression and HFHS feeding on overall LV lysine acetylation was not identified, as overall lysine acetylation remained elevated in the LVs of SIRT3_TG_‐HFHS‐fed mice (Figure 4d). Mitochondrial lysine acetylation was 25% lower in the LV tissue of SIRT3_TG_ mice, compared to WT mice (main effect of diet; *p* = 0.043) (Figure 4e). In HFHS‐fed mice, mitochondrial lysine acetylation was increased by 66% in the LV, compared to control‐fed mice (main effect of diet; *p* = 0.002) (Figure 4e). An interaction between SIRT3 overexpression and HFHS feeding was found for LV mitochondrial lysine acetylation, as mitochondrial lysine acetylation was 27% lower in the LV of SIRT3_TG_‐HFHS‐fed mice when compared to WT‐HFHS‐fed mice (interaction effect; *p* = 0.032) (Figure 4e).

**FIGURE 5:**
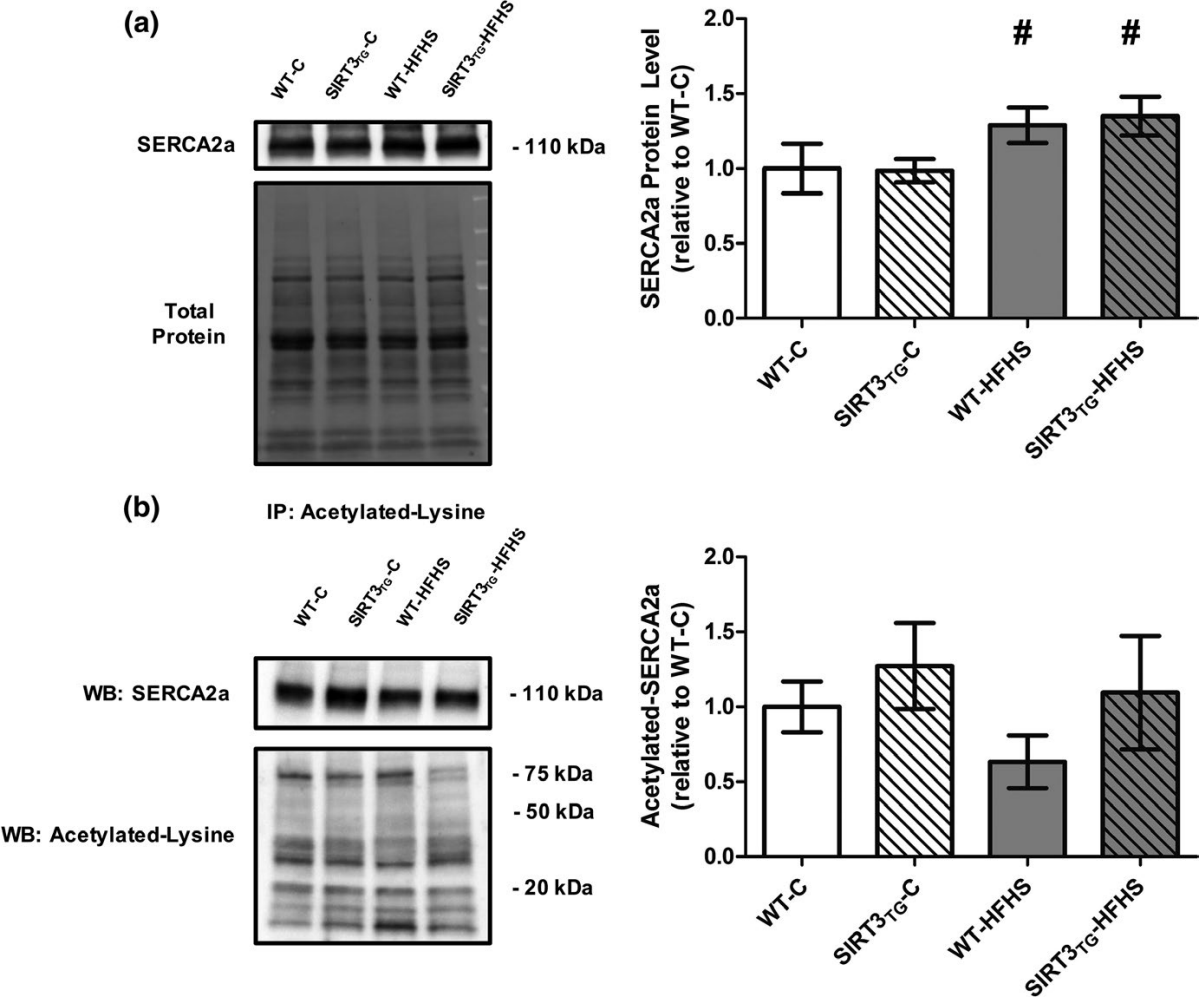
Representative Western blots and graphs depicting LV protein level of SERCA2a and acetylated‐SERCA2a from WT or SIRT3TG mice following 4‐months of control‐ or HFHS‐feeding. (a) SERCA2a. (b) Acetylated‐SERCA2a following immunoprecipitation of acetylated‐lysine containing proteins and detection of SERCA2a by Western blotting. ^#^ indicates a main effect of diet (*p* ≤ 0.05). Compared using a two‐way ANOVA and a Tukey post‐hoc test (SERCA2a: *n* = 10 mice per group; Acetylated‐SERCA2a: *n* = 6 mice per group). Graphs are presented as the mean ± SE

**FIGURE 6:**
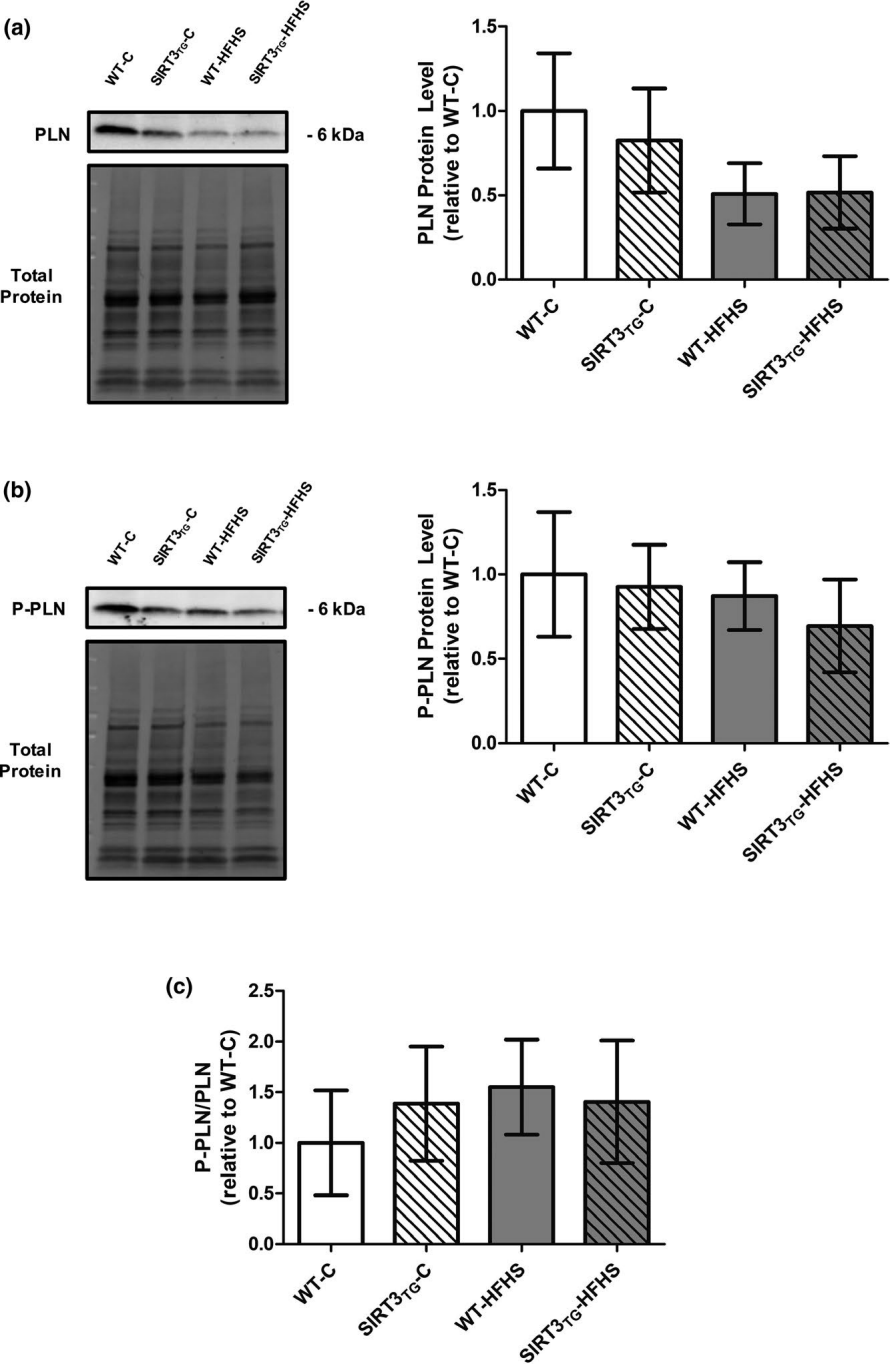
Representative Western blots and graphs depicting LV protein level of PLN, phosphorylated‐PLN, and the phosphorylated‐PLN to total‐PLN ratio from WT or SIRT3TG mice following 4‐months of control‐ or HFHS‐feeding. (a) PLN. (b) Phosphorylated‐PLN. (c) Phosphorylated‐PLN to Total‐PLN ratio. Compared using a two‐way ANOVA and a Tukey post‐hoc test (*n* = 10 mice per group). Graphs are presented as the mean ± SE

**FIGURE 7:**
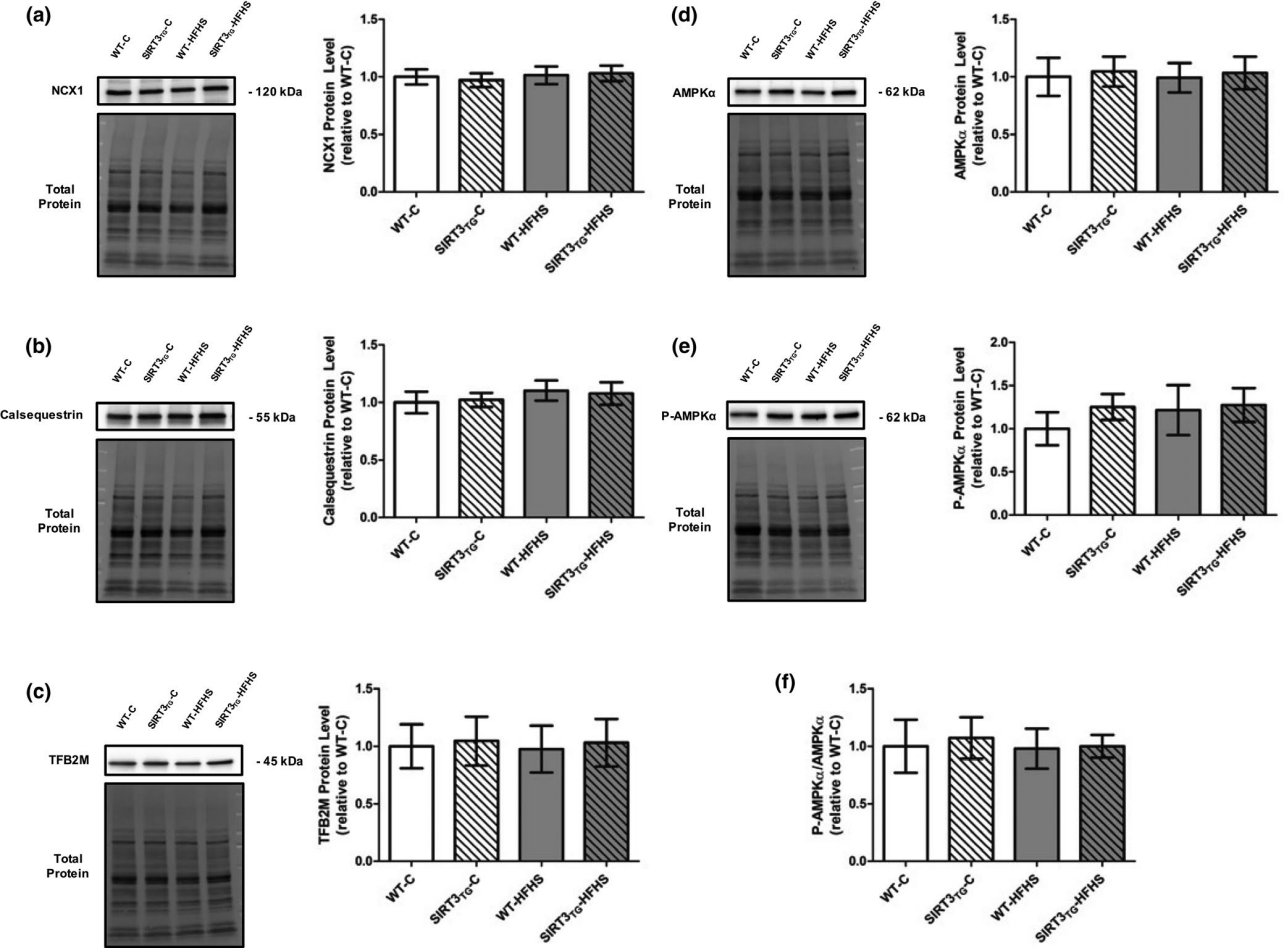
Representative Western blots and graphs depicting LV protein level of NCX, Calsequestrin, TFB2 M, AMPKα, phosphorylated‐AMPKα, and the phosphorylated‐AMPKα to total‐AMPKα ratio from WT or SIRT3TG mice following 4‐months of control‐ or HFHS‐feeding. (a) NCX1. (b) Calsequestrin. (c) TFB2 M. (d) AMPKα. (e) Phosphorylated‐AMPKα. (f) Phosphorylated‐AMPKα to Total‐AMPKα ratio Compared using a two‐way ANOVA and a Tukey post‐hoc test (*n* = 10 mice per group). Graphs are presented as the mean ± SE

Muscle‐specific SIRT3 overexpression did not alter LV SERCA2a protein level (Figure 5a). The protein level of SERCA2a in LV tissue was increased by 33% in HFHS‐fed mice, compared to control‐fed mice (main effect of diet; *p* = 0.014) (Figure 5a). LV SERCA2a acetylation was not altered by the overexpression of SIRT3 or HFHS feeding (Figure 5b). LV PLN and phosphorylated‐PLN protein levels and the phosphorylated‐PLN to total‐PLN ratio were unaltered by SIRT3 overexpression or HFHS feeding (Figure 6). Neither SIRT3 overexpression nor HFHS feeding influenced LV NCX1, Calsequestrin, TFB2 M, AMPKα, and phosphorylated‐AMPKα protein levels, or the phosphorylated‐AMPKα to total‐AMPKα ratio (Figure 7).

### 3.4 Muscle‐specific sirtuin 3 overexpression enhanced cardiac SERCA2a activity

**FIGURE 8:**
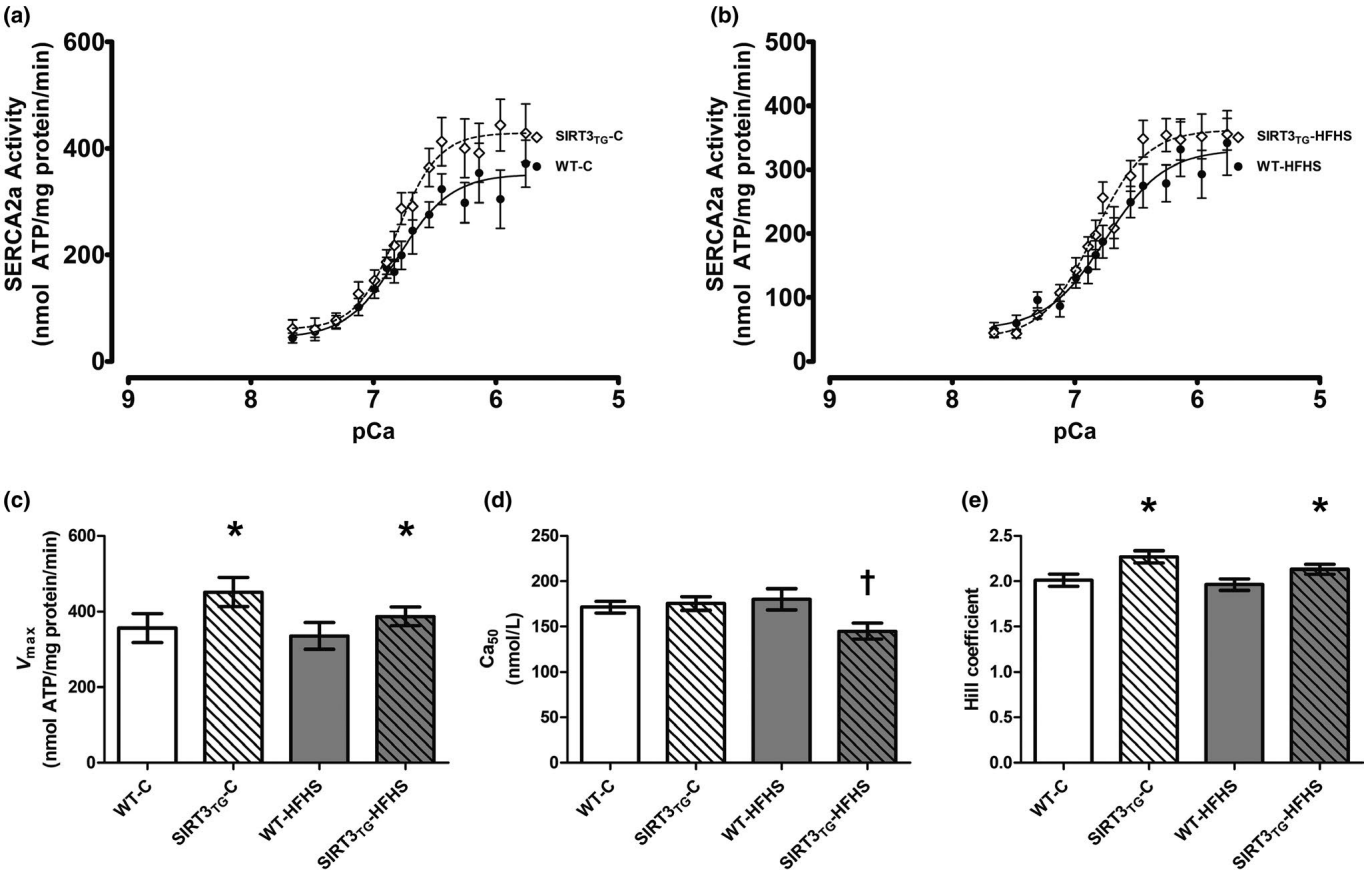
SERCA2a activity and the Ca2+‐dependent kinetic properties of SERCA2a in the LV of WT or SIRT3TG mice following 4‐months of control‐ or HFHS‐feeding. (a) SERCA2a activity‐pCa curves displaying LV SERCA2a ATP hydrolysis over Ca2+ concentrations ranging from a pCa of 7.66 to 5.76 for WT or SIRT3TG mice following 4‐months of control‐feeding. (b) SERCA2a activity‐pCa curves displaying LV SERCA2a ATP hydrolysis over Ca2+ concentrations ranging from a pCa of 7.66 to 5.76 for WT or SIRT3TG mice following 4‐months of HFHS‐feeding. (c) SERCA2a *V*max (maximal enzyme activity). (d) SERCA2a Ca50 ([Ca2+] eliciting 50% of *V*max). (e) SERCA2a Hill coefficient (slope of the relationship between [Ca2+] and enzyme activity for 10%–90% of *V*max). * indicates a main effect of genotype (*p* ≤ 0.05). ^†^ indicates an interaction between genotype and diet (*p* ≤ 0.05). Compared using a two‐way ANOVA and a Tukey post‐hoc test (*n* = 12 mice per group). Graphs are presented as the mean ± SE

SERCA2a ATP hydrolysis was measured over pCa concentrations ranging from 7.66 to 5.76 in vitro using LV tissue homogenates prepared from WT and SIRT3_TG_ mice following either 4 months of control‐ (Figure 8a) or HFHS feeding (Figure 8b). SERCA2a *V*_max_ was 21% higher in the LV tissue of SIRT3_TG_ mice, compared to WT mice (main effect of genotype; *p* = 0.039; Figure 8c). HFHS feeding did not alter SERCA2a *V*_max_ in the LVs of HFHS‐fed mice (Figure 8c). SIRT3 overexpression and HFHS feeding alone did not alter the LV SERCA2a Ca_50_, which is the [Ca^2+^] eliciting 50% of *V*_max_ (Figure 8d). However, an interaction between genotype and diet was identified for the SERCA2a Ca_50_, where the Ca_50_ of SIRT3_TG_‐HFHS‐fed mice was 24% lower, compared to WT‐HFHS‐fed mice, indicating an increase in LV SERCA2a Ca^2+^ sensitivity (interaction effect; *p* = 0.036) (Figure 8D). The Hill coefficient, which quantifies the slope of relationship between SERCA2a activity and [Ca^2+^] for 10%–90% of *V*_max_, was 11% higher in SIRT3_TG_ mice, compared to WT mice (main effect of genotype; *p* = 0.002) (Figure 8e). The Hill coefficient was unaltered by HFHS feeding (Figure 8e).

## 4 DISCUSSION

The results of this study indicate that muscle‐specific SIRT3 overexpression does not attenuate the pathological effects of HFHS feeding in mice, since mice overexpressing SIRT3 in cardiac and skeletal muscle that were fed a HFHS diet displayed similar levels of obesity, glucose intolerance, impaired cardiac function, and adverse cardiac remodeling as WT mice also fed a HFHS diet. These findings suggest that increased SIRT3 protein levels in cardiac and skeletal muscle are not sufficient to prevent diet‐induced obesity, glucose intolerance, or cardiac dysfunction associated with these conditions. The study also sought to determine if HFHS feeding alters cardiac SERCA2a acetylation, and it was revealed that cardiac SERCA2a acetylation is not influenced by HFHS feeding. The final objective of the study was to determine if SIRT3 alters cardiac SERCA2a acetylation and regulates cardiac SERCA2a activity. The results indicate that muscle‐specific SIRT3 overexpression does not alter SERCA2a acetylation in the LV, but SIRT3 overexpression does enhance cardiac SERCA2a activity in mice through a mechanism independent of direct SERCA2a deacetylation.

Diets that are high in fat and sugar increase risk of obesity and T2D, which are conditions that lead to cardiac dysfunction and pathological changes to cardiac structure (Carbone et al., 2015; Zheng et al., 2018). SIRT3 is the primary mitochondrial HDAC, stimulating numerous enzymes involved in cellular metabolism and ATP synthesis through deacetylation (Rardin et al., 2013). In addition to its mitochondrial location, SIRT3 is known to translocate to the nuclear and cytoplasmic compartments of the cell under stress conditions, where it has the ability to deacetylate native proteins (Sundaresan et al., 2008). Therapeutic strategies targeting SIRT3 might, therefore, be useful to prevent the development of obesity and/or T2D in humans. In fact, previous research has reported that SIRT3 is critical to maintain metabolic health in HF‐fed mice (Hirschey et al., 2011). Accordingly, we employed a gain‐of‐function mouse model to determine if muscle‐specific SIRT3 overexpression attenuates the pathological effects of HFHS feeding, as the role of SIRT3 overexpression in conditions of macronutrient excess has not been previously explored. In our study, SIRT3_TG_ mice, which overexpress SIRT3 in cardiac and skeletal muscle, displayed obesity, glucose intolerance, impaired cardiac function, and adverse cardiac remodeling to the same extent as WT mice when both were subjected to 4 months of HFHS feeding. As well, both WT and SIRT3_TG_ mice fed a HFHS diet displayed increased liver weights, potentially resulting from hepatic lipid accumulation. These results reveal for the first time that muscle‐specific SIRT3 overexpression does not attenuate the pathological effects of HFHS feeding. These novel findings are in contrast to the study conducted by Hirschey et al. reporting the rapid development of obesity, insulin resistance, hyperlipidemia, and hepatic steatosis in SIRT3_KO_ mice fed a HF diet (Hirschey et al., 2011). However, although SIRT3 is a necessary regulator of lipid oxidation and glucose metabolism, it is possible that this effect of SIRT3 is not enhanced when it is present at supra‐physiological levels in cardiac and skeletal muscle. Previous research has also found a role for SIRT3 in the context of cardiac hypertrophy, as transgenic mice overexpressing SIRT3 were reportedly protected from the development of angiotensin II‐induced cardiac hypertrophy (Sundaresan et al., 2009). However, angiotensin II treatment leads to greater cardiac dysfunction when compared to the HFHS feeding model we employed, and this may have resulted in increased activation of SIRT3 in mice subjected to angiotensin II treatment because SIRT3 activity depends on cellular stress levels resulting from perturbations to the NAD^+^/NADH ratio (Dolinsky, 2017). This is supported by a previous study which found SIRT3 activation by NAD^+^ is necessary to blunt isoproterenol‐induced cardiac hypertrophy (Pillai et al., 2010).

Lysine acetylation is a common PTM linked to cellular metabolism (Choudhary et al., 2014). We found that overall, as well as mitochondrial specific, lysine acetylation was upregulated in the LVs of both WT and SIRT3_TG_ mice in response to 4 months of HFHS feeding. This finding is in accordance with a previous study by Alrob et al. which reported increased cardiac lysine acetylation in HF‐fed mice (Alrob et al., 2014). The increased lysine acetylation accompanying HFHS and HF diets likely results from metabolic adaptations, such as increased fatty acid utilization and decreased glucose utilization (Alrob et al., 2014). This is because the transition from glycolysis to oxidative metabolism is dependent upon the acetylation of lysine residues located on metabolic intermediates within the mitochondria (Choudhary et al., 2014). We found that SIRT3 overexpression in cardiac muscle did not alter overall lysine acetylation in LV tissue in mice, regardless of diet. This finding contrasts with a previous study using SIRT3_KO_ mice, which found increased overall lysine acetylation in the livers of SIRT3 deficient mice (Hirschey et al., 2011). Though it must be mentioned that our study utilized a muscle‐specific, gain‐of‐function mouse model of SIRT3 overexpression, which fundamentally differs from SIRT3_KO_ mouse models in that SIRT3_KO_ mice completely lack the SIRT3 protein across all tissues; as such, this difference could be explained by tissue‐specific control of lysine acetylation by SIRT3. However, we observed that lysine acetylation was reduced in the LV mitochondrial fractions of SIRT3_TG_ mice, which demonstrates that the overexpressed SIRT3 protein was enzymatically active and capable of deacetylating lysine residues within the cardiac mitochondria. SIRT3 overexpression was also observed to attenuate the rise in cardiac lysine acetylation within the mitochondria in response to HFHS feeding, indicated by lower mitochondrial lysine acetylation in the LV of SIRT3_TG_‐HFHS‐fed mice as compared to WT‐HFHS‐fed mice.

Cardiac SERCA2a activity has been found to be modified by changes to its acetylation status (Gorski et al., 2019; Meraviglia et al., 2018). Previously, Meraviglia et al. demonstrated that increased SERCA2a acetylation was associated with enhanced SERCA2a activity in rat primary cardiomyocytes treated with a HDAC inhibitor (Meraviglia et al., 2018). While Gorski et al. have reported that increased SERCA2a acetylation coincides with decreased SERCA2a activity in the diseased human heart (Gorski et al., 2019). However, it is unknown if HFHS feeding alters cardiac SERCA2a acetylation. Therefore, the next objective of the study was to determine if cardiac SERCA2a acetylation is altered by HFHS feeding. The results of our co‐immunoprecipitation experiments reveal that HFHS feeding does not alter cardiac SERCA2a acetylation. In the study conducted by Meraviglia et al., rat cardiomyocytes were treated with a HDAC inhibitor, which upregulated acetylation of cardiac SERCA2a and also increased cardiac SERCA2a activity (Meraviglia et al., 2018). The differences in SERCA2a acetylation between our study and the study conducted by Meraviglia et al. are likely due to the fact that Meraviglia et al. used an in vitro model where rat primary cardiomyocytes were exposed directly to a HDAC inhibitor; thus, ensuring a high level of lysine acetylation within the HDAC‐treated cardiomyocytes. To date, the only other study examining cardiac SERCA2a acetylation was conducted by Gorski et al., who reported that SERCA2a acetylation was increased in severely diseased cardiac tissue, obtained from humans with heart failure undergoing LV assist device implantation or heart transplantation surgery (Gorski et al., 2019). As such, the cardiac tissue used by Gorski et al. to assess SERCA2a acetylation was significantly more diseased when compared to the mouse LV tissue used in our study. These results seem to suggest cardiac SERCA2a acetylation is not increased in a mouse model of mild diet‐induced cardiac dysfunction and remodeling, absent of heart failure. This observation is supported by our data which reports preserved systolic function and no increase in LV SERCA2a acetylation in HFHS‐fed mice, despite evidence of diastolic dysfunction and adverse cardiac remodeling. Though this observation cannot be conclusively stated because little research has been conducted so far to examine SERCA2a acetylation in the heart.

The SIRT family of proteins may regulate the acetylation status and activity of cardiac SERCA2a. In fact, Gorski et al. additionally reported that SIRT1 deacetylates cardiac SERCA2a and rescues cardiac SERCA2a activity in a mouse model of heart failure induced by transverse aortic constriction (Gorski et al., 2019). Moreover, it has been found that diabetic mice treated with resveratrol, which is a potent SIRT1 activator, exhibit upregulated cardiac SERCA2a activity (Sulaiman et al., 2010). To explore if other SIRT proteins influence cardiac SERCA2a acetylation and activity, we sought to determine if SIRT3 overexpression alters cardiac SERCA2a acetylation and if SIRT3 regulates cardiac SERCA2a activity. Our study found SIRT3 overexpression does not alter cardiac SERCA2a acetylation, as no difference in LV SERCA2a acetylation was identified between SIRT3_TG_ and WT mice. Although we did observe that SIRT3 regulates cardiac SERCA2a activity, as demonstrated by enhanced LV SERCA2a activity in mice overexpressing SIRT3. Specifically, SERCA2a *V*_max_ was increased in the LVs of SIRT3_TG_ mice, indicating SIRT3 overexpression stimulates SERCA2a Ca^2+^ transport in cardiomyocytes. The Hill coefficient, which is the slope of the relationship between SERCA2a activity and Ca^2+^ concentration for 10%–90% of *V*_max_ was increased in SIRT3_TG_ mice, demonstrating an effect of SIRT3 overexpression on SERCA2a enzymatic function. Finally, the SERCA2a Ca_50_ was lower in SIRT3_TG_‐HFHS‐fed mice compared to WT‐HFHS‐fed mice, suggesting increased SERCA2a Ca^2+^ affinity in these mice. In the SIRT3_TG_ mice of our study, the increase in cardiac SERCA2a activity was not accompanied by significant changes in cardiac performance, as measured by echocardiography. However, we did observe a trend of increased SV in SIRT3_TG_ mice as the study progressed. Enhanced cardiac SERCA2a function due to SIRT3 overexpression is a noteworthy result because alterations to SERCA2a function are often detected in the diseased human heart, and therapies that increase cardiac SERCA2a activity may have potential for the treatment of heart disease (Gwathmey et al., 2011, 2013; Jaski et al., 2009).

Prior to conducting the experiment, we speculated that SIRT3 may directly deacetylate SERCA2a since SIRT3 has been reported to translocate from the mitochondria to the cytosol, where retains its deacetylase activity Iwahara et al., 2012). However, we did not observe changes to the acetylation status of cardiac SERCA2a in response to SIRT3 overexpression that would explain the increase in cardiac SERCA2a activity. Thus, the observed regulation of SIRT3 on SERCA2a activity was independent of direct SIRT3‐mediated SERCA2a deacetylation. Moreover, the enhanced SERCA2a activity detected in the LVs of SIRT3_TG_ mice was not a result of increased SERCA2a protein levels in these mice. Based on those observations, it appears that SIRT3 overexpression influences SERCA2a function through yet to be identified mechanism. The study confirmed that SIRT3 overexpression altered cardiac mitochondrial lysine acetylation, but overall lysine acetylation was not altered in the LV tissue; therefore, a mitochondrial‐dependent process may have initiated a signaling pathway that resulted in the activation of cardiac SERCA2a activity. A number of post‐translational modifications, protein‐protein interactions, hormones, and microRNAs (miRNAs) modify SERCA2a function (Stammers et al., 2015). As an example, SERCA2a activity is modulated by PLN, where PLN phosphorylation alters the direct interaction of PLN with SERCA2a to influence Ca^2+^ affinity (SERCA2 Ca_50_), but has little effect on SERCA2a *V*_max_ (MacLennan & Kranias, 2003). However, no changes to PLN or phosphorylated‐PLN protein levels, or the phosphorylated‐PLN to total‐PLN ratio were detected in SIRT3_TG_ mice. That observation indicates SIRT3 overexpression did not enhance SERCA2a activity through the PLN‐mediated control of SERCA2a. However, PLN can also be acetylated (Starling et al., 1996). In fact, PLN‐acetylation has been reported to increase the affinity of PLN for SERCA in skeletal muscle, leading to an approximately 53% decrease in maximal SERCA activity (Starling et al., 1996). It is possible that the overexpression of SIRT3 in the current experiment resulted in the deacetylation of PLN, which may have enhanced cardiac SERCA2a *V*_max_ in the SIRT3_TG_ mice of our study. It is also possible that the observed changes in cardiac SERCA2a activity resulted from mechanisms other than acetylation/deacetylation in SIRT3_TG_ mice. For example, SUMOylation of cardiac SERCA2a occurs when a small ubiquitin‐like modifier type (SUMO1) protein binds to the lysine^480^ and lysine^585^ residues of SERCA2a (Kho et al., 2011). SUMOylation of cardiac SERCA2a increases SERCA2a *V*_max_ and has been found to preserve SERCA2a activity and SERCA2a protein stability in the diseased heart (Kho et al., 2011). Cardiac SERCA2a activity may, therefore, have been enhanced in SIRT3_TG_ mice due to changes in cardiac SERCA2a SUMOylation induced by SIRT3 overexpression. However, we were unable to determine if SIRT3 overexpression altered PLN acetylation/deacetylation or SERCA2a SUMOylation in our study because there was insufficient LV tissue from the study mice to perform further co‐immunoprecipitation experiments. Future research examining such mechanisms and their influence on the regulation of cardiac SERCA2a function will be informative.

Our study explored the effect of muscle‐specific SIRT3 overexpression and HFHS feeding on other SERCA2a regulators. Adenosine monophosphate‐activated protein kinase (AMPK) is an energy sensing protein that is activated by metabolic stress (Shirwany & Zou, 2010). Our group has previously demonstrated that the activation of AMPKα through exercise training increases SERCA2a mRNA expression, protein level, and activity in cardiac and skeletal muscle (Morissette et al., 2014; 2019). In the current study, no changes to AMPKα or phosphorylated‐AMPKα protein levels, or the phosphorylated‐AMPKα to total‐AMPKα ratio were found in SIRT3_TG_ mice, indicating muscle‐specific SIRT3 overexpression did not enhance SERCA2a activity through AMPKα‐activation. Cardiac SERCA2a gene transcription is also regulated by mitochondrial transcription factor B2 (TFB2M) (Watanabe et al., 2011), and we found no difference in TFB2M protein levels between SIRT3_TG_ and WT mice.

Our study revealed that cardiac SERCA2a protein level was increased in mice following 4 months of HFHS feeding. This finding is contrary to previous studies assessing cardiac SERCA2a mRNA expression and protein levels in models of diabetes, which often report downregulation of SERCA2a mRNA and total protein. For example, in animal models of type 1 diabetes (T1D) induced by streptozotocin (STZ), which causes pancreatic β‐cell destruction, hyperglycemia, and ventricular dysfunction, cardiac SERCA2a mRNA expression and protein levels are consistently reported to be diminished (Cheng et al., 2011; Epp et al., 2013; Sulaiman et al., 2010; Trost et al., 2002; Vasanji et al., 2004; Zarain‐Herzberg et al., 1994). Cardiac SERCA2a mRNA expression and protein level are also reduced in Otsuka‐Long‐Evans Tokushima Fatty rats, a genetic model of T2D characterized by hyperglycemia and diastolic dysfunction (Sakata et al., 2006). Further, LV SERCA2a protein level has been reported to be reduced in a model of T1D using Akita^ins2^ mice, which possess a mutation in the insulin 2 gene (LaRocca et al., 2012). However, these studies primarily used T1D animal models or models of advanced T2D, characterized by severe insulin resistance or overt insulin depletion and significant cardiac dysfunction. Using a model of early T2D, Fredersdorf et al. reported an increase in LV SERCA2a mRNA expression and protein level (Fredersdorf et al., 2012). In addition, Fredersdorf et al. found insulin treatment was able to increase SERCA2a mRNA expression in primary rat cardiomyocytes (Fredersdorf et al., 2012). In our study, HFHS feeding led to glucose intolerance, demonstrated by elevated blood glucose concentration following an IPGTT; however, fasting blood glucose concentration was not affected by HFHS feeding. These changes to glucose tolerance, in the absence of elevated fasting blood glucose concentration, suggest our HFHS‐fed mice were in an intermediate stage between normal glucose tolerance and overt T2D (Beulens et al., 2019). Upregulated insulin secretion was likely present at this stage, developing from tissue insulin resistance (Khan et al., 2019). These findings suggest hyperinsulinemia, which occurs during the development of T2D, may stimulate cardiac SERCA2a mRNA expression and protein level.

### 4.1 Limitations

A limitation of our study is that we utilized a transgenic mouse line targeting the muscle‐specific overexpression of the full‐length SIRT3 protein to the mitochondria, as a result of a 25 amino acid mitochondrial localization sequence that is normally found at the N‐terminus of full‐length SIRT3 (Yang et al., 2010). Proteomic screening has identified three acetylation sites on the protein structure of SERCA2a (lysine^464^, lysine^510^, and lysine^533^), all of which reside in its cytoplasmic nucleotide‐binding domain (Foster et al., 2013). Consequently, the acetylation status of cardiac SERCA2a may not have been altered in these SIRT3_TG_ mice because the SIRT3 protein might not have had sufficient access to the cytoplasmic acetylation sites of cardiac SERCA2a. The decision to use this transgenic mouse line was made based on evidence that SIRT3 is stress‐responsive and the full‐length form of SIRT3 translocates from the mitochondria to other cellular compartments, including the cytosol, in response to cellular stress (Iwahara et al., 2012; Sundaresan et al., 2008), potentially providing it access to the cytosolic SERCA2a acetylation sites. As well, our co‐immunoprecipitation experiments did not measure site‐specific cardiac SERCA2a acetylation in SIRT3_TG_ or HFHS‐fed mice. Thus, although overall SERCA2a acetylation was not altered, differential acetylation at the three acetylation sites of SERCA2a could alter SERCA2a activity without changing overall cardiac SERCA2a acetylation in SIRT3_TG_ or HFHS‐fed mice. A final limitation of our study is that we did not perform contractility or Ca^2+^ transient measurements using primary cardiomyocytes isolated from our study mice to determine if the increase in cardiac SERCA2a activity led to relevant changes in cardiomyocyte function in SIRT3_TG_ mice that were not detectable with echocardiography. The isolation of primary cardiomyocytes from the adult mouse heart and subsequent measurements of contractile force and Ca^2+^ transients is possible, but technically challenging (Callaghan et al., 2020).

### 4.2 Future directions

Given that SIRT3 overexpression enhanced cardiac SERCA2a activity without directly deacetylating the SERCA2a protein, future studies should be conducted to identify the mechanism or pathway through which SIRT3 overexpression regulates cardiac SERCA2a activity. Furthermore, future studies could be conducted to determine if stimulating SIRT3 expression or activation in cardiac muscle has the capacity to enhance SERCA2a activity and cardiac performance in the diseased heart.

### 4.3 Conclusion

Muscle‐specific SIRT3 overexpression does not attenuate the pathological effects of HFHS feeding in mice. This study also revealed that the acetylation status of cardiac SERCA2a is not altered by HFHS feeding or SIRT3 overexpression. Despite that, SIRT3 overexpression enhanced cardiac SERCA2a activity by a mechanism independent of direct SERCA2a deacetylation. Finally, an increase in cardiac SERCA2a protein level in HFHS‐fed mice was observed. The results of this study add to the existing literature examining the biological relevance of SIRT proteins in disease states and characterizes cardiac SERCA2a acetylation/deacetylation in a mouse model of mild diet‐induced cardiac dysfunction and remodeling. These findings indicate that SIRT3 overexpression in cardiac muscle enhances SERCA2a activity in the mouse heart.

## CONFLICTS OF INTEREST

The authors declare no conflicts of interest.

## AUTHOR CONTRIBUTIONS

T. A. D. conceived and designed the research. V. W. D provided transgenic animals. C. J. O., T. L. M., and B. X. performed experiments. C. J. O. analyzed data. C. J. O., K. A. O., and T. A D. interpreted results of experiments. C. J. O. prepared figures and drafted the manuscript. C. J. O. and T. A. D. edited and revised the manuscript. All authors approved the final version of the manuscript.
